# Evaluation of the Impact of Different Management Methods on *Tetranychus urticae* (Acari: Tetranychidae) and Their Predators in Citrus Orchards

**DOI:** 10.3390/plants11050623

**Published:** 2022-02-25

**Authors:** Amine Assouguem, Mohammed Kara, Hamza Mechchate, Fahd A. Al-Mekhlafi, Fahd Nasr, Abdellah Farah, Abderahim Lazraq

**Affiliations:** 1 Laboratory of Functional Ecology and Environment, Faculty of Sciences and Technology, Sidi Mohamed Ben Abdellah University, Fez 30000, Morocco; lazraqab@gmail.com; 2 Laboratory of Applied Organic Chemistry, Faculty of Sciences and Technology, Sidi Mohamed Ben Abdellah University, Fez 30000, Morocco; farah.abdellah1@gmail.com; 3 Laboratory of Biotechnology and Conservation and Valorization of Natural Resources (LBCVRN) (Ex LBPRN), Sidi Mohamed Ben Abdellah University, Fez 30000, Morocco; mohammed.kara@usmba.ac.ma; 4 Laboratory of Inorganic Chemistry, Department of Chemistry, University of Helsinki, P.O. Box 55, FI-00014 Helsinki, Finland; 5 Department of Zoology, College of Science, King Saud University, Riyadh 11451, Saudi Arabia; falmekhlafi@ksu.edu.sa; 6 Department of Pharmacognosy, College of Pharmacy, King Saud University, Riyadh 11451, Saudi Arabia; fahdnasr74@gmail.com

**Keywords:** *Tetranychus urticae*, *Euseius stipulatus*, *Typhlodromus* sp., *Phytoseiulus persimilis*, citrus orchard, monitoring, predators, pest, treatments

## Abstract

To evaluate the effectiveness of eco-friendly treatments based on detergents classified as non-hazardous and black soap on the pest *Tetranychus urticae* Koch 1836, and their predators (*Euseius stipulatus* Athias-Henriot, 1960, *Typhlodromus* sp., *Phytoseiulus persimilis* Athias-Henriot, 1957), different treatments were applied to citrus orchards planted with Valencia late (Orange) in the Mechraa Belksiri region of Morocco (T0 = control experiment; T1 = spirodiclofen 0.5 L/Ha; T2 = 125 L/Ha (5%) of black soap; T3 = detergent; 4 L/Ha of Oni product + 2 L/Ha of Tide product). The results obtained during the whole monitoring period indicated that the three treatments used, namely spirodiclofen, black soap, and detergents, ensured a reduction in the rate of population of the pest *T. urticae* compared to the untreated plot. In the untreated plot, the average was 45.01 A± 4.90 mobile forms, while the plot treated with spirodiclofen it was only 21.10 C ± 2.71, the black soap 31.49 B ± 3.35, and in the plot treated with detergents, the average was similar to that obtained by spirodiclofen (22.90 C ± 2.18). On the predators (*E. stipulatus*, *P. persimilis*, and *Typhlodropmus* sp.), the black soap and the treatment with detergents were less harmful compared to the chemical spirodiclofen.

## 1. Introduction

Citrus is one of the world’s major fruit crops and is grown in over 100 countries [1]. It belongs to the Rutaceae family, with 140 genera and 1300 species, including fundamental groups like orange, lemons, mandarin, and pummelos. They are cultivated in tropical and subtropical areas [2]. The total worldwide production of citrus is 139.80 million tons [3].

In Morocco, thanks to the Ministry of Agriculture, citrus orchards have reached 130,000 ha with a total annual production of over 2.2 million tons [4]. However, the yield at the national level is still low compared to that achieved by other European and American countries [5]. The Ministry of Agriculture is currently trying to remedy constraints that are hampering the increase of citrus production, including lack of manpower, maturity of orchards, and biotic and abiotic constraints [6,7]. The negative impact caused by pests reduces the quantity and quality of production. Indeed, spider mites, aphids, medflies, and diaspine scales are pests of primary economic importance [8,9]. When conditions are favorable and in the absence of adequate methods of control, significant damage is often observed on the fruits, twigs, leaves, and young shoots of citrus [10]. *Tetranychus urticae* Koch, 1836 is an economically major pest to agricultural crops and ornamental plants throughout the world [11], being found in Europe, Asia, Africa, the Caribbean islands, and North America [12]. It is known for developing a resistance to pesticides [13], and causes significant damage to agricultural crops, such as defoliation, leaf yellowing, and leaf burning [14,15]. *Euseius stipulatus* Athias-Henriot, 1960 (Acari: Phytoseiidae), *Phytoseiulus persimilis* Athias-Henriot, 1957 (Acari: Phytoseiidae), and *Typhlodromus* sp. (Acari: Phytoseiidae) are the main predators of *T. urticae* in many regions of the world [16,17]. Spirodiclofen (Envidor 240 SC, Bayer, SA) is an acaricide of the keto-enol family. It acts by contact and ingestion as an inhibitor of lipid biosynthesis (LBI) [18]. It has a powerful ovicidal action, controls all juvenile stages, and significantly reduces fertility in females [19].

Black soap, brown in color, is biodegradable, non-polluting, and an excellent insecticide [20]. This product is effective against insects like mealy bugs, aphids, whiteflies, thrips, and spider mites. Through simple contact, it blocks the respiratory pores [2,21]. It does not produce toxic residues and does not affect natural predators. These products are approved by organic agriculture (EEC regulation 2092/91). To minimize the negative impact of these pests on citrus production, the use of chemical applications is often the solution adopted by growers [22,23]. Spirodiclofen often gives good results in the control of spider mites, namely, the biological parameters, including developmental time, survival rate, and in particular, the fecundity of *T. urticae* [24,25]. The misuse of this active ingredient often has harmful impacts on the agroecosystems [26,27]. The development of new eco-friendly approaches is a necessity. In this context, our study aims to evaluate the impact of spirodiclofen, black soap, and a mixture of two detergents (Oni and Tide products) on the pest *T. urticae* and its predators (*E. stipulatus*, *Typhlodromus* sp., and *P. persimilis*).

## 2. Materials and Methods

### 2.1. Study Area

**Figure 1:**
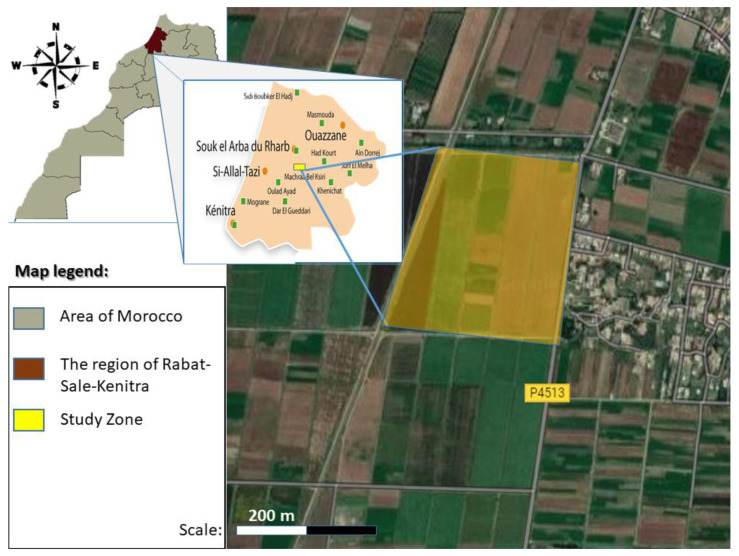
Location of experimental orchards.

This study was performed in Mechra Bel Ksiri, located north of Oued Sebou in the Gharb region, at an altitude of 300–500 m above sea level (Figure 1) [28]. The climate is temperate to hot. This region is well known for the production of citrus fruits, cereals, and vegetable crops due to the suitable properties of the climate and soil [29]. The Gharb region is characterized by a Mediterranean climate with annual precipitation ranging between 480 and 600 mm/year, and an average temperature of 27 °C in summer and 13 °C in winter [30].

### 2.2. Sampling Design

**Figure 2:**
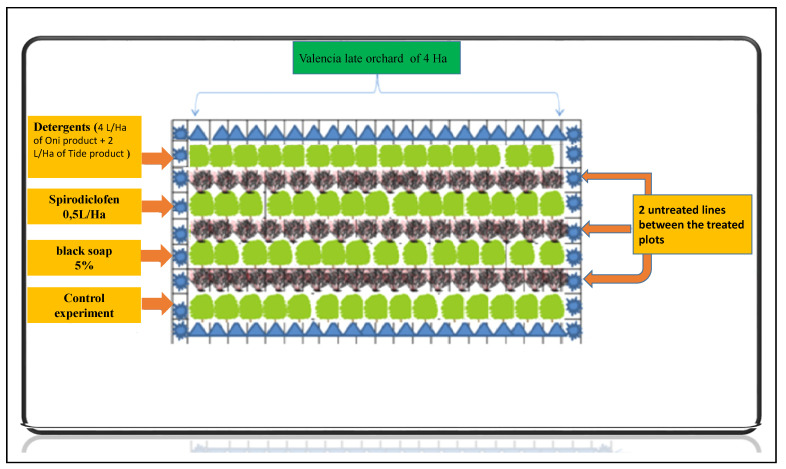
The different types of treatments used in the citrus orchard.

A Valencia late (*Citrus sinensis*) orchard with a high spider mite infestation was selected to study the impact of spirodiclofen, black soap, and a mixture of two detergents (Oni product and Tide product) on *Tetranychus urticae* populations and their predators. Spirodiclofen was used because of its effectiveness against a wide range of biting/sucking pests, including spider mites. The orchard covers 4 ha of Valencia late trees. The orchard was divided into 4 plots of 1 ha (Figure 2), and each plot was treated with a specific dose of the treatment: (i) T0 treated with water only (as a control experiment), (ii) T1 = spirodiclofen 0.5 L/Ha, which is the recommended dose for treating citrus mites, (iii) T2 = 125 L/Ha (5%) of black soap, and (iv) T3 = detergent 4 L/Ha of Oni product (sodium C14–17 alkyl sec sulfonate, sodium C12–13 pareth sulfate) and 2 L/Ha of Tide product (sodium C10–16 alkylbenzene sulfonate, sodium borate, and propylene glycol). These two detergents are often used by local farmers to control pests and minimize the use of pesticides. They are not classified as hazardous, according to the European directive 99/45/EC on dangerous preparations [31]. The treatments were applied with the Teyme Eolo sprayer (Teyme Tecnologia Agricola, Girona, Spain), with turbulent nozzles 12 mm in diameter, delivering 1.55 L/min at 20 bar pressure. The towed sprayer delivers 2500 L of spray liquid per hectare, at a rate of 6 L of spray solution for each tree (Figure 2). To evaluate the effect of each product used, a block of 10 trees was selected and monitored weekly. Ten leaves were collected from each tree, from different directions (north, east, south, and west) and at different heights of the tree (10 replicates were performed independently) [32,33,34]. We left two untreated lines (12 m) between the different treated plots to avoid overlapping treatments. The total number of predators (*P. persimilis, Typhlodromus* sp., and *E. stipulatis*) and phytophagous mite (*T. urticae*) found on the 10 leaves of each plot was recorded [35]. All mites, except eggs and mites in quiescence, were counted on both sides of each leaf with a professional eye loop 10×. Then, to confirm the number of mites on each leaf, the collected leaves were transferred directly into polyethylene bags referenced to the laboratory for observation under a binocular microscope. Inspections were conducted 3 days after the treatment: 12 April (week 1), 19 April (week 2), 26 April (week 3), 3 May (week 4), 10 May (week 5), 17 May (week 6), 24 May (week 7), and 1 June (week 8).

### 2.3. Statistics

Statistics of the data were performed using Minitab software, version 1.1.19, Minitab, Sydney, NSW, Australia. The results were given as percentage and mean ± SD. In order to evaluate the effectiveness of the treatments used on the pests and predators, we calculated the average of mobile forms found of *T. urticae* in (T0 = control experiment; T1 = plot treated with spirodiclofen; T2 = plot treated with black soap; T3 = plot treated with a mixture of two detergents). In parallel, we evaluated the impact of the treatments on natural enemies. We calculated the averages of each predator (*P. persimilis*, *Typhlodromus* sp. and *E. stipulatis*) in T0, T1, T2, and T3 [36]. We tested for normality and homogeneity of variance for all variables with the Kolmogorov–Smirnov test. The impact of treatments, monitoring dates, and their interactions was compared using the general linear model (GLM) univariate, followed by a post hoc Tukey test at *p* < 0.05. The principal component analyses (PCA) were accomplished using Minitab 19.1 software (Minitab, State College, PA, USA) to elucidate the relationship between the different mites studied and the treatments tested.

## 3. Results

### 3.1. Leaf Occupancy Rate by Mites in the Different Plots

During the whole monitoring period, on the untreated plot, the occupancy of the inspected leaves by the pest *Tetranychus urticae* was *n* = 3601 mobile forms (54%); moreover, the predator *Typhlodromus* sp. presented the most important proportion with 18% (*n* = 1212), followed by *P. persimilis* with 15% (*n* = 1032) and *E. stipulatus* with 13% (*n* = 838).

In the plot treated with spirodiclofen, the abundance of these mites was low compared to the untreated plot. The pest *T. urticae* presented 46% with 1688 mobile forms. The predator *P. persimilis* presented 22% (*n* = 805), while *E. stipulatus* and *Typhlodromus* sp. presented the same proportion of 16% (*n* = 595 and *n* = 571, respectively).

In the plot managed with black soap, we recorded 2519 mobile forms (50%) for the pest *T. urticae*, 19% (*n* = 948) for the predator *P. persimilis*, 17% (*n* = 832) for *Typhlodromus* sp., and only 14% (*n* = 728) for *E. stipulatus*.

**Figure 3:**
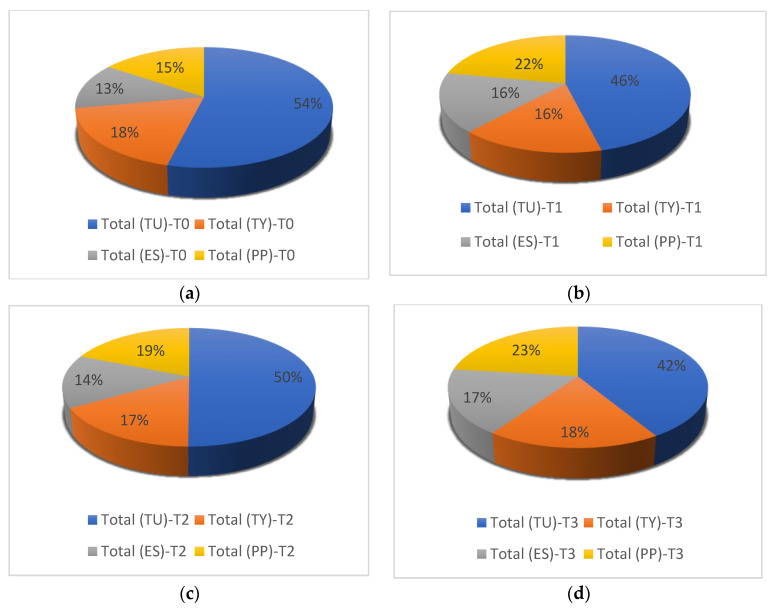
The proportion of the different mites studied (the phytophagous mite *Tetranychus urticae* (TU) and their predators *Euseius stipulatus* (ES), *Typhlodromus* sp. (TY), and *Pytoseiulus persimilis* (PP)) according to the treatments used (T0 = control experiment; T1 = spirodiclofen 0.5 L/Ha; T2 = 125 L/Ha (5%) of black soap; T3 = 4 L/Ha of Oni product + 2 L/Ha of Tide product). (**a**) Control experiment; (**b**) Spirodiclofen 0.5 L/Ha; (**c**) Black soap 125 L/Ha; (**d**) T3 = Mixture of two detergents.

In the plot treated with detergents, we recorded 42% (*n* = 1832) of *T. urticae*, 23% (*n* = 1022) of *P. persimilis*, 18% (*n* = 809) of *Typhlodromus* sp., and 17% (*n* = 766) of *E. stipulatus* (Figure 3).

### 3.2. The Influence of Treatments on the Different Mites Studied

**Figure 4:**
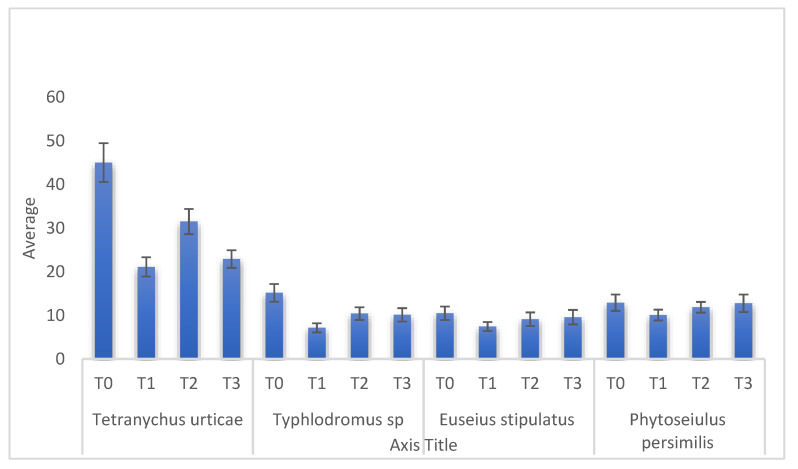
Comparison between the means of different mites according to the treatments (T0 = control experiment; T1 = spirodiclofen 0.5 L/Ha; T2 = 125 L/Ha (5%) of black soap; T3 = detergents; 4 L/Ha of Oni product + 2 L/Ha of Tide product).

Regarding the effect of the different treatments used on the mites studied, the treatment with detergents and spirodiclofen showed the highest efficiency on the populations of the pest *T. urticae* (Figure 4), while on the predators (*E. stipulatus*, *P. persimilis*, and *Typhlodropmus* sp.), the black soap and the treatment with detergents were less harmful compared to the chemical product (spirodiclofen). Table 1 confirms these results; spirodiclofen treatment significantly reduced *T. urticae* populations with a mean of 21.10 C ± 2.71 compared to the control (45.01 A ± 4.90). The same result was observed with the detergent treatment, which reduced the *T. urticae* levels to an average of 22.90 C ± 2.18. Black soap provided a significant reduction for the mean of this pest (31.49 B ± 3.35) compared to the control; however, this treatment was less effective on *T. urticae* compared to the treatment with spirodiclofen and detergents.

The highest average of *Typhlodromus* sp. was in the water treatment, with an average of 15.15 A ± 1.83 and the treatment with black soap (T3) and detergents (T4) were less harmful on the predator *Typhlodromus* sp. with the means of 10.40 B ± 2.05 for the plot treated with black soap and 10.11 B ± 2.14 for the one sprayed with detergents, while the treatment with spirodiclofen (T2) had the most harmful impact on the population of *Typhlodromus* sp., with an average of 7.13 C ± 1.28.

For the predator *E. stipulates,* the average was 10.47 A ± 1.63 in the plot treated with water (T0), while it was 9.10 AB ± 1.25 in the plot treated with black soap (T3), and 9.57 AB ± 1.47 in the plot treated with detergents (T4). The average of this population was low in the plot treated with the chemical product spirodiclofen, with a mean of 7.44 B ± 1.37.

On *P. persimilis* populations, the treatment with detergents was the least harmful, with an average (14.68 A ± 1.87) nearly similar to that for the plot treated only with water (16.02 A ± 2.12). With black soap, the average was 11.85 AB ± 1.11, whereas the plot treated with spirodiclofen was 10.06 B ± 1.15.

**Figure 5:**
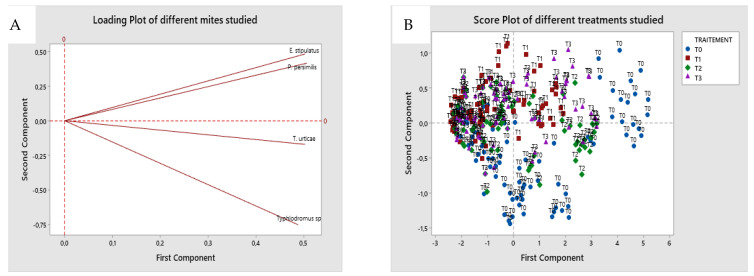
Principal component analysis (PCA) of the different mites studied (*T. urticae*; *E. stipulatus*; *Typhlodromus* sp.; *P. persimilis*) according to the different treatments (T0 = control experiment; T1 = spirodiclofen 0.5 L/Ha; T2 = 125 L/Ha (5%) of black soap; T3 = detergents; 4 L/Ha of Oni product + 2 L/Ha of Tide product) (**A**); double projection diagram for the two components (**B**).

In Figure 5, the eigenvalues of the first two principal components explain 92.7 % of the variation in the data. The first principal component explains 86.5% of the total variance. The variables with the highest association with the first principal component (CP1) are *T. urticae* (0.50), *E. stipulatus* (0.50), and *P. persimilis* (0.50), while the variable *Typhlodromus* sp. (−0.75) is negatively associated with the second principal component (CP2). However, the projection of the scoring and the contribution diagram can visually show a positive contribution of the four mites studied (*T. urticae, P. persimilis, Typhlodromus* sp. and *E. stipulatis*) on the first main axis in positive correlation with T0 water treatment, while spirodiclofen (T2), detergents (T4), and black soap (T3) treatment were correlated negatively.

### 3.3. Fluctuation of Mites According to Treatments and Follow-Up Dates

**Figure 6:**
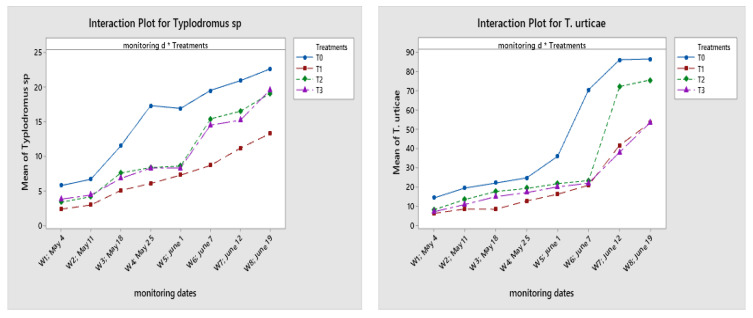

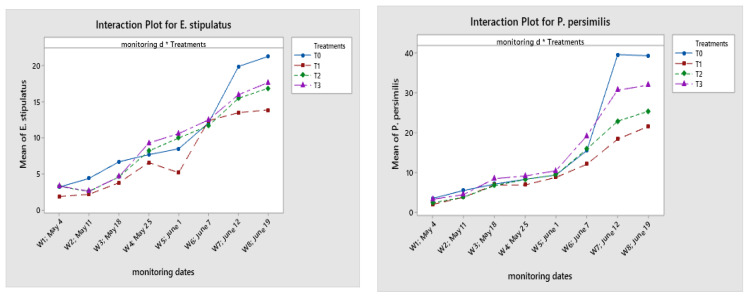
Variation of the means of *T. urticae* and their predators (ES: *E. stipulatus*; TY: *Typhlodromus* sp.; PP: *P. persimilis*) in the four plots (T0 = control experiment; T1 = spirodiclofen 0.5 L/Ha; T2 = 125 L/Ha (5%) of black soap; T3 = detergent; 4 L/Ha of Oni product + 2 L/Ha of Tide product) according to the interaction between different monitoring dates and treatments (d = dates).

According to the results in Table 2, we can conclude that the interaction between the treatment used and the monitoring dates has a significant effect on the variation of the means of the different mites studied. During the first week, three days after the spraying of the treatments, we observed a significant decrease in the rate of *T. urticae*; we had an average of 6.30 F ± 1.16 for the spirodiclofen, 8.20 E ± 1.69 for the black soap (Table 3), and 07.00 F ± 1.49 for the detergent treatment. In contrast, in the untreated plot T0, and during the same week (W1), this average almost doubled 14.30 E ± 2.00. Despite the increase in temperature from 27 to 34 °C from week 1 to week 5 (Table 3), the rate of *T. urticae* had a slight increase during the first five weeks after treatment with spirodiclofen compared to the average reached in the untreated plot (Figure 6). In week 5, these averages were 16.30 D ± 1.41 in the plot treated with spirodiclofen and 36.00 C ± 2.35 in the untreated plot. Beyond that, this pest had a significant increase during the last three weeks to reach an average of 53.60 A ± 4.77 in the plot treated with spirodiclofen (T1), and 86.60 A ± 7.06 in the untreated plot during week 8, where the temperature reached 39 °C.

For the treatment based on detergents, we obtained similar results to those achieved with spirodiclofen. These products ensured a significant reduction of the pest *T. urticae* during the eight-week monitoring period. Despite the increase in temperature from 27 °C to 38 °C (Table 4), the treatment with black soap ensured a significant decrease in the rates of *T. urticae* during the first six weeks after application compared to the untreated plot (Figure 6). During the sixth week, these averages were 23.40 B ± 1.43 on the plot sprayed with black soap and 70.60 B ± 4.09 on the untreated plot. The number of this pest increased during the last 2 weeks of monitoring (weeks 7 and 8) to reach 75.70 A ± 4.69 in week 8, almost the same as the average reached on the untreated plot (86.60 A ± 7.06). 

During the whole monitoring period, Figure 6 indicates that the three treatments used caused a decrease in the means of *T. urticae* from 12 April (week 1) to 19 May (week 8) compared to the untreated plot; the effectiveness of spirodiclofen was important compared to the control by black soap and detergents.

Regarding the interaction between treatments and monitoring dates (Figure 6), all three treatments used had a significant impact on the abundance of *Typhlodromus* sp. over time. This influence was greater in the spirodiclofen than the detergent and black soap treatments.

For *Typhlodromus* sp., this difference was less important during the first five weeks after spraying the treatments. On the date of 18 May, these averages were 7.30 CD ± 1.41 in T1 (spirodiclofen), 8.60 C ± 1.49 in T2 (black soap), and 8.30 C ± 1. 30 in T3 (detergents). Then, there was a significant difference between the treatment with spirodiclofen and the treatments with black soap and detergents from the sixth week until the last week. The averages were 8.70 BC ± 1.45 in T1, 15.40 B ± 2.14 in T2, and 14.50 B ± 1.94 in T3.

During the first five weeks, the detergent and black soap treatments showed less harmful impacts compared to spirodiclofen on the *E. stipulatus* population. The averages at this time (1 June) were 5.20 B ± 1.41 in T1 (spirodiclofen), 10.00 BC ± 1.29 in T2 (black soap), and 10.60 BC ± 1.71 in T3 (detergents).

For *P. persimilis,* the detergent treatment showed almost similar results as in the control, and showed the least harmful impact, in the last three weeks of the follow-up (from 7 to 19 June). In the plot treated with detergents, we had an average of 32.0 A ± 2.97 for this species; in the untreated plot, we had 39.30 A ± 2.87; in the plot treated with black soap, the average was 25.90 A ± 2.87; and the average was 21.60 A ± 2.04 in the one treated with spirodiclofen.

## 4. Discussion

The biodiversity of phytoseiid mites nationally and internationally has started to decline catastrophically, as well as the increase in resistance developed by the pests against acaricides, allowing unusual increases in the population of the latter. This work aims to compare the impact of detergents, black soap, and spirodiclofen on the pest *T. urticae* on the one hand and on phytoseiid mites on the other hand.

The results collected during the study period showed that the three treatments used, namely, spirodiclofen, black soap, and detergents, ensured a decrease in the population rate of the pest *T. urticae* compared to the untreated plot. In the untreated plot, the average was 45.01 A ± 4. 90 mobile forms, while the plot treated with spirodiclofen was only 21.10 C ± 2.71, the black soap gave 31.49 B ± 3.35, and average of the plot treated with detergents was similar to that obtained by spirodiclofen at 22.90 C ± 2.18. The same results were reported by [26,37]: the application of spirodiclofen ensured the decrease of the survival rate and fecundity of *T. urticae.* In another study, the efficacy of spirodiclofen and other acaricides against *T. urticae* on greenhouse strawberries was tested at different times after application. At seven days, spirodiclofen showed significant efficacy at 86.6%. This efficacy reached 90% on day 10 and started to decrease slightly after that period [38]. In addition, black soap also showed significant efficacy against several insects such as *Bemisia tabaci* Gennadius, 1889, *Aphis craccivora* Koch, 1854 and *parlatoria ziziphi* Lucas, 1853 [39,40]. The pesticidal activity of the agricultural detergents SU-120 and Tecsa^®^ Fruta was evaluated on *T. urticae* and *Myzus persicae* Sulzer, 1776. Several concentrations of SU-120 were applied to nymphs and adults of *T. urticae*. Mortality was measured in both cases at 24 h. On *T. urticae*, both detergents caused mortality greater than 70% [41,42,43].

This study revealed that all three treatments used had an impact on the predators studied compared to the control, with the influence of spirodiclofen being greater than that of the detergents and black soap treatments. The effect of spirodiclofen was studied on *Neoseiulus californicus* McGregor, 1954, a potential predator of *T. urticae*, and the results indicated that the adverse consequences on the population growth parameters were significant [44]. In a small field plot test on spirodiclofen-treated apple trees, its use in a conventional chemical control program affected populations of *Forficula auricularia* Linnaeus, 1758 (Dermaptera), which is an important generalist predator in orchards, regulating populations of several damaging pest species, while the number of earwigs increased in the orchards with the compatible spray program (Integrated Pest Management) [45]. Many studies confirm that black soap and detergents can replace conventional chemical treatments and constitute an important tool in integrated pest management because of their low phytotoxicity [43,46].

The results revealed that there was an impact of the treatments used on the three predators compared to the control, and this influence was greater with the spirodiclofen than with the detergents and black soap treatments. Despite the benefits of pesticides, such as the rapidity of action in the reduction of number of pests and their easy use when compared to natural extracts from plants [16], chemical control has many limitations; the examination of the action spectrum of the active components used throughout the world reveals that 46% of acaricides and 72% of insecticides are globally toxic towards auxiliary arthropods and public health [47]. Concerning the impact of the treatments according to the dates on *T. urticae*, we noticed that the three treatments ensured a decrease in the abundance of this pest. On the other hand, the efficacy of spirodiclofen was superior to the black soap and detergents; this can be explained by the high toxicity of the pesticide [22], as it can be remedied by monthly repetitive spraying with black soap or/and detergents, which remain less toxic for the plant, the predators, and the agroecosystem in general [39]. Botanical pesticides from the Lamiaceae, the Asteraceae, the Myrtaceae, and the Apiaceae taxons can also be used as a complementary alternative in the control of *T. urticae* [48]. We can explain the increase of the number of pests during the last two weeks of follow-up by the increase in the temperature, which remains a paramount parameter for population fluctuation [49,50].

## 5. Conclusions

The following conclusions can be drawn from this work: the black soap and detergent products can replace conventional chemical treatments and are an important tool in integrated pest management, provided they are used correctly. The repetitive spraying of eco-friendly products can ensure the reduction of the pest rates in the long term. Finally, the release of natural enemies can be ensured after four weeks of the treatment with these products, since their persistence does not exceed five weeks, according to our results.

## Figures and Tables

**Table 1:** The impact of different treatments used on the mites studied (TU: *T. urticae*; ES: *E. stipulatus*; TY: *Typhlodromus* sp.; PP: *P. persimilis*) in citrus orchards. Values in the same column with different superscripts are significantly different (*p* < 0.05).

	TU	TY	ES	PP
Control experiment (T0)	45.01 ^A^ ± 4.90	15.15 ^A^ ± 1.83	10.47 ^A^ ± 1.63	16.02 ^A^ ± 2.12
Spirodiclofen 0.5 L/Ha (T1)	21.10 ^C^ ± 2.71	7.13 ^C^ ± 1.28	7.44 ^B^ ± 1.37	10.06 ^B^ ± 1.15
Black soap 5% (T2)	31.49 ^B^ ± 3.35	10.40 ^B^ ± 2.05	9.10 ^AB^ ± 1.25	11.85 ^AB^ ± 1.11
Detergent (T3)	22.90 ^C^ ± 2.18	10.11 ^B^ ± 2.14	9.57 ^AB^ ± 1.47	14.68 ^A^ ± 1.87

**Table 2:** General linear model (GLM) of analysis of variance for the mean density of *T. urticae* (**a**), *Typhlodromus* sp. (**b**), *P. persimilis* (**c**), and *E. stipulatus* (**d**) according to the treatments, monitoring dates, and their interactions.

**(a) *T. urticae***					
**Source**	**DF**	**Adj SS**	**Adj MS**	**F-Value**	***p*-Value**
Monitoring dates	7	135.717	19,388.1	1579.57	0.000 *
Treatments	3	28.572	9523.9	775.92	0.000 *
M. dates * Treatments	21	18.865	898.4	73.19	0.000 *
Error	288	3535	12.3		
Total	319	186.689			
**(b) *Typhlodromus* sp.**					
**Source**	**DF**	**Adj SS**	**Adj MS**	**F-Value**	***p*-Value**
Monitoring dates	7	7966.9	1138.13	211.42	0.000 *
Treatments	3	2634.3	878.11	163.12	0.000 *
M. dates * Treatments	21	579.6	27.60	5.13	0.000 *
Error	288	1550.4	5.38		
Total	319	12,731.2			
**(c) *P. persimilis***					
**Source**	**DF**	**Adj SS**	**Adj MS**	**F-Value**	***p*-Value**
Monitoring dates	7	30,048	4292.56	473.97	0.000 *
Treatments	3	1745	581.70	64.23	0.000 *
M. dates * Treatments		3012	143.44	15.84	0.000 *
Error	288	2608	9.06		
Total	319	37,413			
**(d) *E. stipulatus***					
**Source**	**DF**	**Adj SS**	**Adj MS**	**F-Value**	***p*-Value**
Monitoring dates	7	8996.3	1285.19	304.76	0.000 *
Treatments	3	389.7	129.90	30.80	0.000 *
M. dates * Treatments		409.6	19.50	4.62	0.000 *
Error	288	1214.5	4.22		
Total	319	11,010.1			

* Statistically significant *p*-values (*p* *<* 0.05).

**Table 3:** Fluctuation of the different species studied (TU: *T. urticae*; ES: *E. stipulatus*; TY: *Typhlodromus* sp.; PP: *P. persimilis*) in the four treated plots (T0 = control experiment; T1 = spirodiclofen 0.5 L/Ha; T2 = 125 L/Ha (5%) of black soap; T3 = detergents; 4 L/Ha of Oni product + 2 L/Ha of Tide product) according to the different monitoring dates (W: week).

	TU-TO	TU-T1	TU-T2	TU-T3	TY-T0	TY-T1	TY-T2	TY-T3	ES-T0	ES-T1	ES-T2	ES-T3	PP-T0	PP-T1	PP-T2	PP-T3
W1	14.30 ^E^ ± 2.00	6.30 ^F^ ± 1.16	8.20 ^E^ ± 1.69	07.00 ^F^ ± 1.49	5.80 ^D^ ± 1.33	2.40 ^F^ ± 0.63	3.40 ^D^ ± 1.57	3.80 ^D^ ± 1.39	3.20 ^D^ ± 1.81	1.90 ^D^ ± 0.94	3.30 ^D^ ± 1.25	3.30 ^D^ ± 1.25	3.50 ^D^ ± 1.90	2.00 ^D^ ± 1.16	2.40 ^E^ ± 1.02	3.20 ^D^ ± 1.41
W2	19.50 ^DE^ ± 1.51	8.60 ^F^ ± 1.71	13.50 ^D^ ± 2.17	10.80 ^F^ ± 2.39	6.70 ^D^ ± 1.14	3.00 ^EF^ ± 1.04	4.20 ^D^ ± 1.24	4.40 ^D^ ± 1.24	4.40 ^CD^ ± 1.64	2.20 ^D^ ± 1.31	2.60 ^D^ ± 1.07	2.60 ^D^ ± 1.07	5.50 ^CD^ ± 1.44	3.80 ^D^ ± 1.16	3.80 ^DE^ ± 1.34	4.40 ^D^ ± 1.22
W3	22.10 ^D^ ± 2.13	8.50 ^F^ ± 1.35	17.70 ^CD^ ± 4.08	14.90 ^E^ ± 4.33	11.50 ^C^ ± 1.43	5.10 ^DE^ ± 1.37	7.60 ^C^ ± 1.53	6.80 ^CD^ ± 1.56	6.70 ^CD^ ± 1.54	3.80 ^CD^ ± 1.44	4.60 ^D^ ± 1.95	4.60 ^D^ ± 1.53	7.10 ^CD^ ± 1.93	6.90 ^C^ ± 1.53	6.70 ^CD^ ± 1.37	8.40 ^C^ ± 1.43
W4	24.80 ^D^ ± 1.47	12.80 ^E^ ± 1.22	19.30 ^BC^ ± 2.22	17.0 ^DE^ ± 2.87	17.30 ^B^ ± 1.87	6.10 ^CD^ ± 1.10	8.40 ^C^ ± 1.43	8.30 ^C^ ± 1.47	7.70 ^C^ ± 1.70	6.60 ^BC^ ± 1.67	8.20 ^C^ ± 1.22	9.30 ^C^ ± 1.82	8.30 ^C^ ± 1.40	6.90 ^B^ ± 1.89	8.30 ^C^ ± 1.35	9.10 ^C^ ± 1.33
W5	36.00 ^C^ ± 2.35	16.30 ^D^ ± 1.41	21.80 ^BC^ ± 3.15	20.0 ^CD^ ± 2.44	16.90 ^B^ ± 1.79	7.30 ^CD^ ± 1.41	8.60 ^C^ ± 1.49	8.30 ^C^ ± 1.30	8.50 ^BC^ ± 1.87	5.20 ^B^ ± 1.41	10.00 ^BC^ ± 1.29	10.60 ^BC^ ± 1.71	9.40 ^C^ ± 1.59	8.80 ^B^ ± 1.91	9.40 ^C^ ± 1.67	10.40 ^C^ ± 1.4
W6	70.60 ^B^ ± 4.09	21.00 ^C^ ± 1.33	23.40 ^B^ ± 1.43	21.90 ^C^ ± 1.79	19.50 ^A^ ± 1.84	8.70 ^BC^ ± 1.45	15.40 ^B^ ± 2.14	14.50 ^B^ ± 1.94	12.10 ^B^ ± 1.54	12.40 ^A^ ± 1.26	11.70 ^B^ ± 1.82	12.50 ^B^ ± 1.78	15.50 ^B^ ± 2.04	12.10 ^A^ ± 2.01	15.90 ^B^ ± 2.04	19.20 ^B^ ± 1.7
W7	86.20 ^A^ ± 6.42	41.70 ^B^ ± 3.71	72.30 ^A^ ± 5.29	38.00 ^B^ ± 1.29	20.90 ^A^ ± 2.84	11.20 ^AB^ ± 2.44	16.50 ^AB^ ± 2.84	15.20 ^B^ ± 2.35	19.90 ^A^ ± 2.19	13.50 ^A^ ± 1.78	15.50 ^A^ ± 1.54	16.00 ^A^ ± 1.24	39.60 ^A^ ± 2.94	18.40 ^A^ ± 2.56	22.90 ^A^ ± 2.91	30.70 ^A^ ± 2.4
W8	86.60 ^A^ ± 7.06	53.60 ^A^ ± 4.77	75.70 ^A^ ± 4.69	53.60 ^A^ ± 3.77	22.60 ^A^ ± 2.77	13.30 ^A^ ± 1.17	19.10 ^A^ ± 1.27	19.60 ^A^ ± 2.55	21.30 ^A^ ± 2.75	13.90 ^A^ ± 1.83	16.90 ^A^ ± 1.96	17.70 ^A^ ± 1.57	39.30 ^A^ ± 2.87	21.60 ^A^ ± 2.04	25.90 ^A^ ± 2.87	32.0 ^A^ ± 2.97

Values in the same column with different superscripts are significantly different (*p* < 0.05).

**Table 4:** Temperatures recorded during the monitoring period.

Follow-Up Date	4 May	11 May	18 May	25 May	1 June	7 June	12 June	19 June
Temperature	27	28	30	32	34	38	37	39

## Data Availability

Data available upon request.
